# Optimization of NiMo alloy nanoparticles for catalytic dry reforming of methane

**DOI:** 10.3762/bjnano.17.84

**Published:** 2026-08-31

**Authors:** Azimet A Karluk, Javeed Mahmood, Seok-Jin Kim, Cafer T Yavuz

**Affiliations:** 1 Advanced Membranes & Porous Materials (AMPM) Platform and KAUST Catalysis Platform, Oxide & Organic Nanomaterials for Energy & Environment (ONE) Laboratory, Division of Physical Science and Engineering, King Abdullah University of Science and Technology, Thuwal 23955-6900, Saudi Arabia

**Keywords:** bimetallic active sites, heterogeneous catalysis, low carbon syngas, molybdenum doping, nickel nanoparticles, NOSCE mechanism

## Abstract

Metallic Ni nanoparticles doped with Mo are recently developed highly active nanocatalysts for dry reforming of methane (DRM). However, the nature of interactions and co-existence of Ni and Mo, optimal Ni/Mo ratios, as well as the impact of synthetic methods on catalyst structure and activity, remain unclear. This study compares two different synthetic approaches, one-pot synthesis (OP) and post-modification (PM), to determine the optimal Ni/Mo ratio for catalytic performance. The OP method promotes the formation of NiMo nanoparticles under adequate reducing conditions, significantly enhancing DRM activity. In contrast, the PM method ensures precise Ni/Mo ratios but leads to the formation of large MoO_3_ nanocrystals that obstruct active sites, thus reducing the catalytic performance. Our findings demonstrate that the highest DRM activity is achieved with a 10:1 molar ratio of Ni/Mo to predominantly form alloy nanoparticles and emphasize that the synthesis method, along with the stoichiometric ratios, plays a crucial role in achieving exceptional catalytic performance.

## Introduction

Carbon dioxide (CO_2_) is a major greenhouse gas and a carbon reservoir [1–2]. In order to mitigate the role of CO_2_ in climate change, reducing CO_2_ emissions by large scale utilization pathways is crucial [3]. A viable approach is the dry reforming of methane (DRM), which reacts CO_2_ and methane (CH_4_) to generate synthesis gas (syngas, mixtures of CO + H_2_), a valuable resource for energy generation and sustainable chemicals [4]. While the endothermic reaction requires significant energy input for efficient conversion (Equation 1) [5], it could also be considered as an effective way to store excess renewable energy, e.g., syngas battery.


[1] $${\text{CO}}_{2}\;+\;{\text{CH}}_{4}\to 2{\text{H}}_{2}\;+\;2\text{CO }(\Delta H{{}^{\circ}}_{298\text{K}}\;=\;247.5\text{ kJ }\cdot \;{\text{mol}}^{-1})$$

Although DRM offers a promising path for large-scale CO_2_ utilization, its efficiency relies heavily on available catalysts. Noble metals, such as Ru, Rh, Pt, and Pd, have excellent conversion rates and resistance to deactivation with minimal coke formation [6]; however, their high cost and scarcity hinder industrial-scale deployment. Nickel, a non-noble metal, provides a more economical alternative but is susceptible to rapid deactivation due to carbon deposition and sintering [7]. To address these limitations, Ni-based catalysts can be improved by incorporating various supports and promoters [8–9].

Among the various catalytic promoters [10–11], molybdenum has proven particularly effective in enhancing the activity and stability of Ni-based catalysts. Mo helps mitigate nickel oxidation, promotes the dispersion of active Ni sites, and reduces carbon deposition [12–14]. Several studies have shown that an optimal Mo content boosts methane conversion and minimizes coke formation [15–16]. Zhou et al. [15] identified a Ni/Mo ratio of 10:1 as optimal through DFT calculations and experimental testing; our study complements theirs by focusing on different aspects, specifically, the influence of synthesis strategy, systematic variation of Mo content, and the experimental evidence for alloy formation and MoO*_x_* crystallization. In our previous studies, we demonstrated that NiMo nanocrystals supported on single-crystalline (SC) MgO achieved sustained DRM performance for over 850 h without observable coking [17–18]. The exceptional stability is attributed to the migration of particulates to the high-energy step edges of the SC MgO, a process now known as “nano-catalysts on single crystal edges (NOSCE)”. The NOSCE phenomenon ensured the formation of stable and well-dispersed active catalytic sites during the reaction. A critical question on optimal Ni/Mo ratios over SC MgO, however, remains and deserves further investigation.

Zhang et al. reported that NiMo alloys enhance DRM efficiency by improving the activation of CH_4_ and CO_2_ [19]. The presence of Mo promotes the formation of a NiMo alloy, which helps stabilize metallic Ni, maintaining its activity throughout the DRM reaction [19]. Similarly, Wang et al. noted in their theoretical study that the Ni_4_Mo catalyst displays a stronger affinity for intermediates compared to pure Ni, leading to better activity in DRM [20]. However, other studies indicate that adding Mo could decrease DRM activity due to reduced catalyst alkalinity, the formation of the Ni_4_Mo phase, and weak interactions between the metal and support [14]. These conflicting findings emphasize the need to evaluate systematically whether NiMo alloy formation enhances DRM activity and, ultimately, the need to identify the optimal Ni/Mo ratio for maximizing catalytic performance.

In this study, we prepared NiMo nanocatalysts supported on SC MgO with six different Ni/Mo ratios to assess the impact of Mo content on catalytic activity and stability during DRM. We compared two synthetic methods, namely, one-pot synthesis (OP), where Ni and Mo precursors are reduced simultaneously (OP-*x*) [17], and post-modification (PM), which allows for precise control of Mo loading on Ni/MgO (PM-*x*). Our goal was to clarify how alloyed NiMo and residual Mo species bound to Ni on MgO affect catalytic performance and stability. Although Mo loading is more efficient through PM, the one-pot preparation method resulted in superior catalytic activity. Despite the lower activity of PM-*x*, it still exhibited better coke resistance compared to pure Ni on MgO. Coverage of high-energy surface sites by Mo species (or MoO*_x_*) on defective MgO accounts for the effect, demonstrating NOSCE behavior [17,21], which causes carbon species to avoid accumulating on MoO*_x_* surfaces instead of Ni [7,22]. Among the OP-*x* samples, the one with the highest NiMo alloy density and fewer Mo species showed the highest conversion activity, further supporting the hypothesis that excess Mo species alongside the NiMo alloy negatively impact catalytic activity.

## Results and Discussion

**Figure 1:**
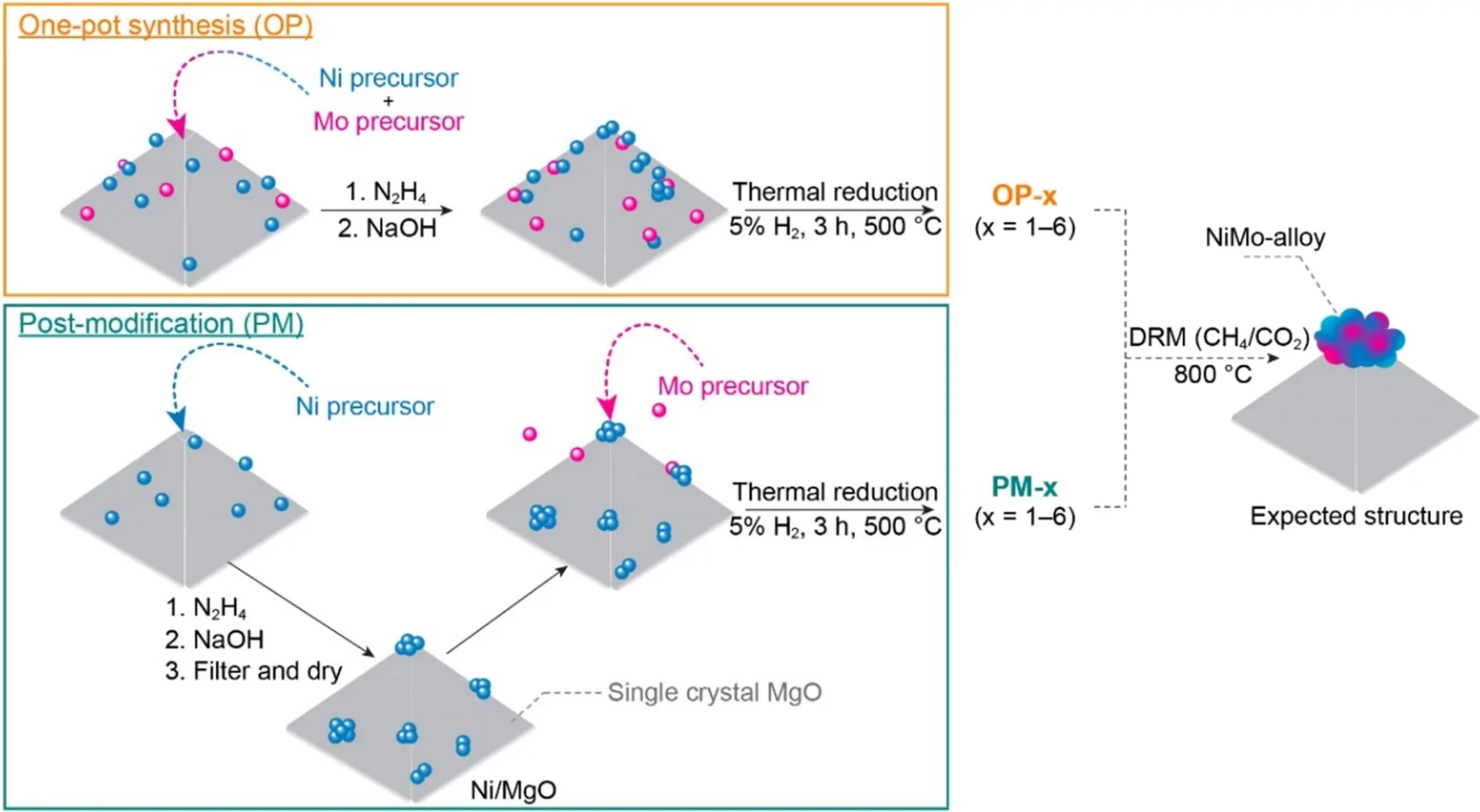
Schematic illustration of NiMo on MgO nanocatalysts’ (OP-*x* and PM-*x*) preparation. Blue spheres represent Ni precursors, pink spheres indicate Mo precursors, and the grey cubic edges depict single-crystalline MgO. Six different Ni/Mo molar ratios were prepared for sample numbers *x* = 1–6, corresponding to theoretical loading Ni/Mo molar ratios of 8.2, 4.1, 2.7, 2.0, 1.6, and 1.3 for OP-*x* and PM-*x*, respectively.

One-pot synthesis (OP-*x*) samples (where the sample number is denoted by *x* = 1–6) were made using nickel chloride hexahydrate (NiCl_2_·6H_2_O) and ammonium molybdate tetrahydrate ((NH_4_)_6_Mo_7_O_24_·4H_2_O) as precursors, with SC MgO serving as the support (Figure 1). The mixture was quickly reduced using hydrazine and sodium hydroxide (NaOH) at 80 °C. Subsequently, the freshly prepared OP-*x* samples underwent an additional reduction in a tube furnace under a 5% H_2_ flow at 500 °C for 3 h, followed by filtration and vacuum drying. In contrast, post-modification (PM-*x*) samples (*x* = 1–6) were prepared by combining fresh Ni on MgO powder with a highly concentrated ammonium molybdate tetrahydrate/ethylene glycol solution, followed by the same thermal reduction procedures. The catalysts with different Ni/Mo molar ratios are labeled as OP-*x* and PM-*x*. For PM-1 through PM-6, the corresponding theoretical loading molar ratios of Ni/Mo were found to be 8.2, 4.1, 2.7, 2.0, 1.6, and 1.3, respectively.

**Figure 2:**
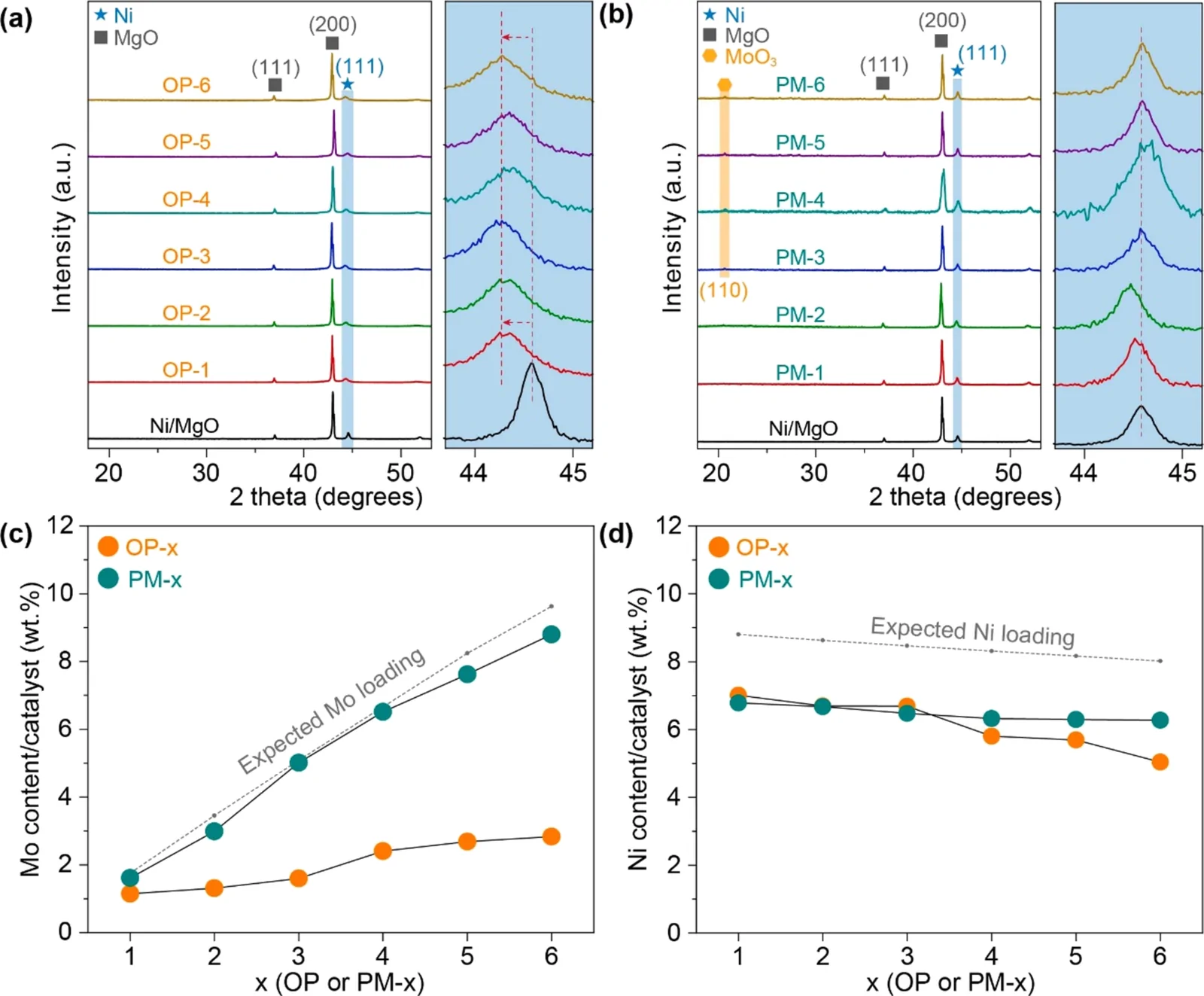
Characterization of NiMo on MgO nanocatalysts. Powder XRD patterns of (a) OP-*x* and (b) PM-*x*, Ni (111) peak exhibit a shift to lower angles as Mo loading increases. (c) Comparison of Mo loading (wt %) determined by ICP-OES and expected loading amounts (wt %) for OP-*x* and PM-*x* series. (d) Comparison between actual Ni loading (wt %) determined by ICP-OES and expected loading amounts (wt %) for both OP-*x* and PM-*x*.

In powder X-ray diffraction (XRD) patterns of OP-*x* and PM-*x* series, peaks corresponding to SC MgO at 37.1°, 43.1°, 62.3°, 74.6°, and 78.6°, confirm stable nanocrystalline structures following the thermal treatment process (Figure 2 and Supporting Information File 1, Figure S1). The samples exhibited characteristic Ni peaks at 44.5°, 51.8°, and 76.4°, corresponding to the (111), (200), and (220) planes, respectively. Compared with the PM series, the Ni(111) diffraction peak in the OP series progressively shifts toward lower 2θ values with increasing Mo loading, indicating effective incorporation of Mo into the Ni lattice and the resulting lattice expansion. In contrast, the PM series exhibits only a slight initial peak shift at low Mo loading, followed by a shift back toward higher 2θ values with further Mo addition, suggesting the segregation of excess Mo as MoO*_x_*, as evidenced by the appearance of additional orthorhombic MoO_3_ diffraction peaks, and limited alloy formation at higher Mo contents [23].

To verify whether the metals were effectively reduced and successfully loaded onto MgO as intended for each synthesis method, the metal contents were quantified using inductively coupled plasma-optical emission spectrometry (ICP-OES). The expected Ni and Mo contents were calculated by accounting for an approximate 10 wt % loss from hydroxy groups on the MgO surface caused by deposited moisture (Supporting Information File 1, Figure S2). The ICP-OES results (Figure 2c,d and Supporting Information File 1, Table S1) showed that PM-*x* catalysts closely matched the intended Mo loading, whereas OP-*x* samples exhibited insufficient loading, especially at elevated Mo contents. The Ni content displayed a moderate decrease as the Mo content increased because the Ni loading was fixed in the procedure. The Ni content of the PM-*x* samples decreased by approximately 0.55%. However, in the case of the OP-*x* series, the Ni content generally followed the expected decreasing trend, fluctuating between 5 and 7 wt %, which can be attributed to differences in reduction dynamics between the two methods.

**Figure 3:**
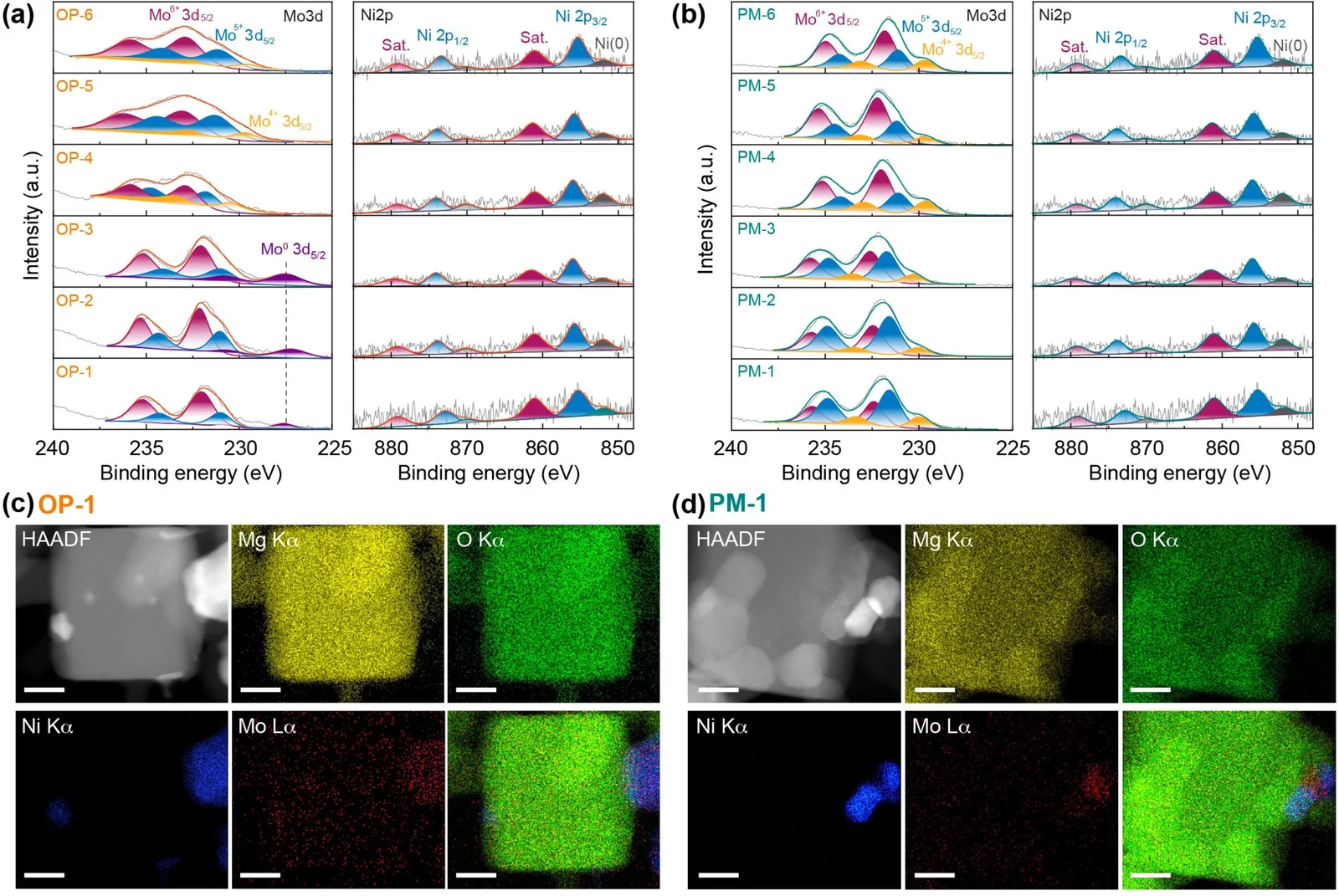
Structural characterization of OP-*x* and PM-*x* nanocatalysts: Mo 3d and Ni 2p XPS spectra of as-synthesized (a) OP-*x* and (b) PM-*x*. High-angle annular dark-field (HAADF) and electron dispersive X-ray (EDX) elemental mappings of (c) OP-1 and (d) PM-1 (scale bar: 50 nm).

X-ray photoelectron spectroscopy (XPS) analysis further highlighted the oxidation states of Mo and Ni in both catalysts (Figure 3). The intense peaks at 855.5 and 873.5 eV correspond, respectively, to Ni 2p_3/2_ and Ni 2p_1/2_, while the peaks at 861.0 and 879.2 eV are satellite peaks, representing shake-up peaks on the higher binding energy side of, respectively, the Ni 2p_3/2_ and Ni 2p_1/2_ edges [24]. Furthermore, the minor peaks at 852.0 and 870.0 eV are attributed to metallic Ni [25].

In the case of the OP-*x* nanocatalysts, as the Mo content increased (beyond OP-3), the Ni 2p_3/2_ and Ni 2p_1/2_ peaks shifted to higher binding energies (857.1 and 875.0 eV, respectively). The observed shift indicates a loss of electron density in metallic Ni, likely due to interactions with Mo species, which promotes the formation of NiMo*_x_*O*_y_* and Ni+NiMo*_x_*O*_y_* (as suggested by the presence of Ni^0^ peaks in all OP-*x* samples) rather than forming a NiMo alloy with excess Mo. It is also worth noting that, while XPS reveals multiple oxidation states of Mo, the XRD patterns indicate that MoO_3_ is the most thermodynamically stable phase present under our synthesis and reaction conditions. These observations are consistent with previously reported findings [26] and are further supported by the shifts in the peaks of Mo^5+^ (231 and 234.3 eV) and Mo^6+^ (232.6 and 235.7 eV) toward higher binding energies as the Mo/Ni ratio increased. Electron transfer from Ni to MoO*_x_* species likely causes the observed shift, reflecting strong Ni–MoO*_x_* interactions [27]. Furthermore, from OP-3 to OP-6, the intensity of the Mo^0^ peak at around 227.3 eV significantly decreases, indicating the progressive formation of NiMo*_x_*O*_y_*, which leads to an increased intensity of Mo^5+^ (231 and 234.3 eV) and Mo^6+^ (232.6 and 235.7 eV) peaks. This suggests that Mo is predominantly present in the Mo^5+^ and Mo^6+^ oxidation states once a specific Mo content threshold is exceeded.

In the PM-*x* nanocatalysts, Mo exists primarily in the oxidation states Mo^4+^ (230 and 233.4 eV), Mo^5+^ (231.6 and 234.9 eV), and Mo^6+^ (232.4 and 235.7 eV), which matched the crystalline MoO_3_ peaks in the XRD patterns at higher Mo/Ni ratios (Figure 2b). The presence of Mo^4+^ peaks (230 and 233.4 eV) in PM-*x*, while being absent in OP-*x*, indicates a less effective reduction in the PM method.

High-resolution transmission electron microscopy (HRTEM) images, selected-area electron diffraction patterns, energy-dispersive X-ray spectroscopy (EDX), and elemental fraction data for OP-2 (Supporting Information File 1, Figure S3) and PM-2 (Supporting Information File 1, Figure S4) confirm the successful deposition of Ni on SC MgO and the co-existence of Ni and Mo. High-angle annular dark-field (HAADF) imaging and the corresponding scanning transmission electron microscopy (STEM) elemental mapping further illustrate the arrangement of NiMo on the SC MgO catalysts. Specifically, the distribution of Mo within the Ni particles, as observed in Figure 3c, demonstrates that alloy formation occurred in the OP-1 catalyst, consistent with previous findings [17]. In contrast, Figure 3d reveals that, in the PM-1 sample, Ni and Mo remain separate, supporting our hypothesis about the differing reduction and integration mechanisms of the two synthesis methods. Although some regions show a partial overlap of Ni and Mo signals, this does not contradict our conclusion; rather, it indicates that MoO*_x_* deposits can form in close proximity to Ni particles without generating a true NiMo alloy. The interpretation aligns with XPS results, which show multiple Mo oxidation states, suggesting the coexistence of different Mo species and the formation of various structures, including amorphous MoO*_x_* (Mo^4+^, Mo^5+^), NiMoO_4_-type phases, Mo^6+^ in MoO_3_, and lower-valent Mo species interacting with Ni to form NiMo alloys. However, SEM and HRTEM analyses revealed the co-existence of NiMo alloy and crystalline MoO_3_ phases, indicating phase segregation despite the presence of multiple oxidation states, as suggested by XPS.

**Figure 4:**
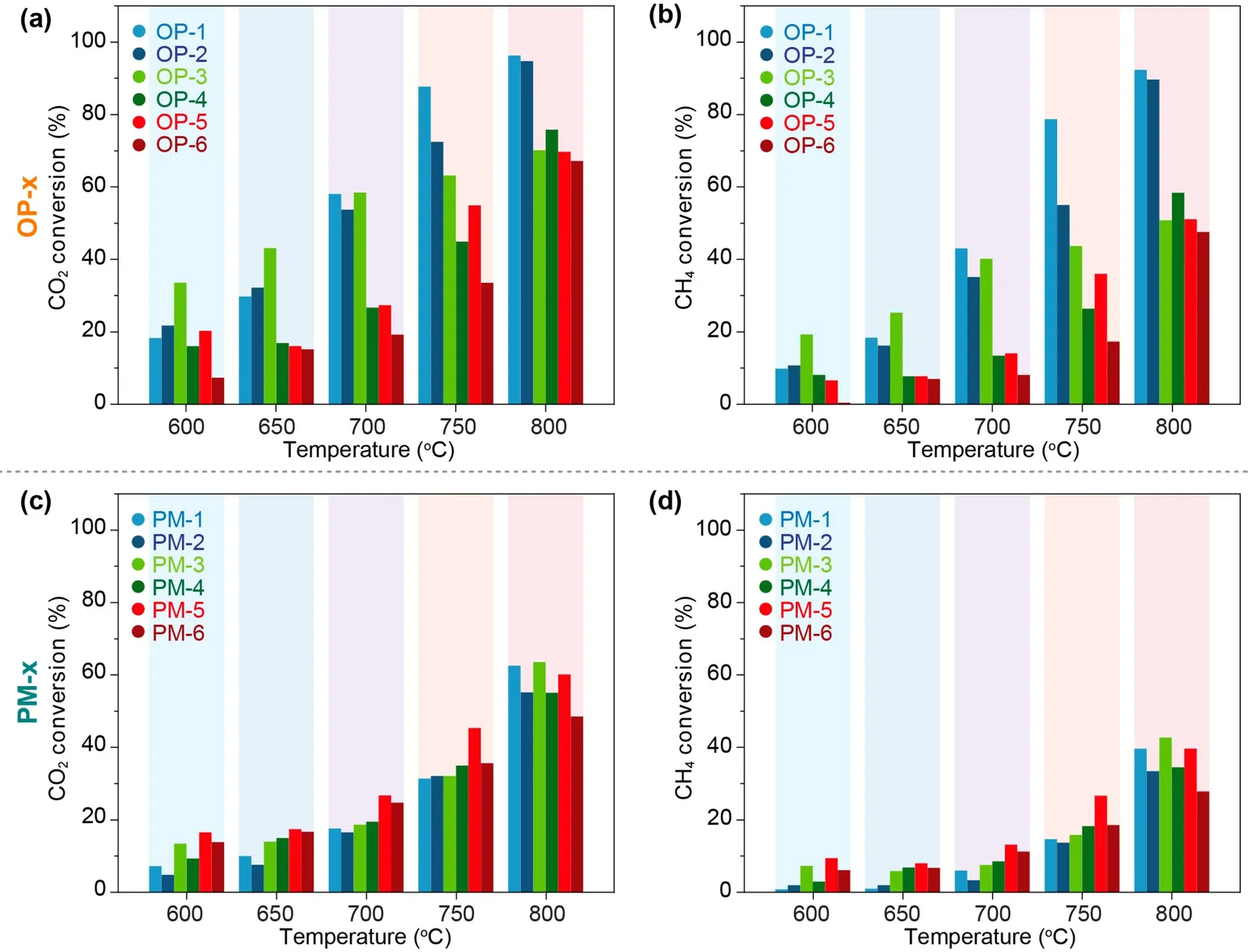
Catalytic performance evaluation of the OP-*x* and PM-*x* nanocatalysts. CO_2_ and CH_4_ conversion yields with (a, b) OP-*x* and (c, d) PM-*x* nanocatalysts at varying reaction temperatures. Reaction conditions: 1 bar, 25 mg OP-*x* or (PM-*x*), CH_4_/CO_2_/N_2_ = 2:2:6, weight hourly space velocity (WHSV) = 25,680 mL·g^−1^·h^−1^.

To clarify the role of Mo species in the OP-*x* and PM-*x* nanocatalysts, the catalytic activity at different temperatures for the DRM reaction was evaluated (Figure 4 and Supporting Information File 1, Figure S5). OP-1 exhibited the highest conversion efficiency at 800 °C, achieving conversions of 96.2% CO_2_ and 92.2% CH_4_. It is also noteworthy that in OP-1, the conversion rates increased after reaching the highest temperature (800 °C), with conversions exceeding those observed before the peak temperature (Figure 4a and Supporting Information File 1, Figure S5). Such behavior indicates that the catalyst underwent activation at higher temperatures, aligning with earlier reports [17].

Additionally, at 800 °C, OP-2 showed a slightly lower but comparable conversion efficiency to that of Ni/MgO (Supporting Information File 1, Figure S6). The results are consistent with theoretical studies indicating that Mo doping promotes initial CH_4_ activation, thereby easing methane dehydrogenation compared with bare Ni(111) [20]. Beyond OP-2, the conversion performance drops significantly as Mo content in the OP samples increases. At lower temperatures (600–650 °C), OP-3 contains additional metallic Mo (Figure 3a), which promotes NiMo alloy formation and enhances catalytic activity. Elevated temperatures result in complex interactions between the MgO support and excess Mo salts or MoO_3_ deposits originating from incomplete reduction. These effects hinder active site accessibility and cause the irregular performance observed in higher Mo-loaded catalysts like OP-5.

**Figure 5:**
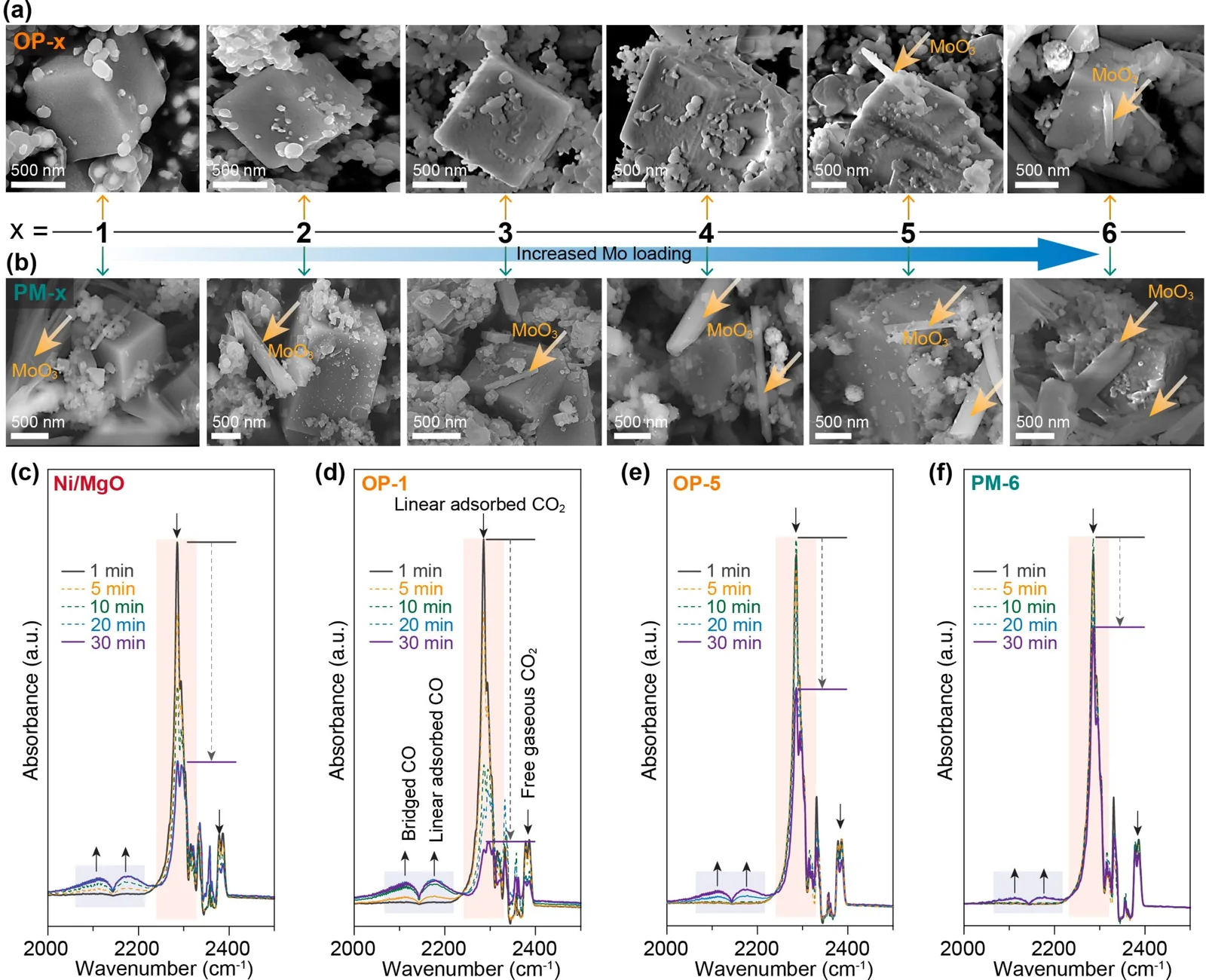
SEM images of (a) OP-*x* samples after DRM and (b) PM-*x* samples before DRM, showing the morphological differences with increasing Mo loading. (c–f) In situ DRIFT spectra collected during CO_2_ reduction at 750 °C on Ni/MgO, OP-1, OP-5, and PM-6, respectively. The spectra were acquired sequentially under pure CO_2_ flow (99.999%, 10 mL·min^−1^, 30 min) followed by pure H_2_ flow (99.999%, 10 mL·min^−1^, 30 min). The OP-1 catalyst exhibits a faster depletion of carbonate-related surface species and more intense CO vibrational signals compared to Ni/MgO, OP-5, and PM-6, indicating enhanced CO_2_ activation and reduction performance.

The presence of a single Mo atom on the Ni surface promotes CO_2_ activation, while multiple Mo atoms slightly reduce the activation efficiency [28]. Moreover, among the reactions involving CH*_x_* oxidation and CH*_x_*O dehydrogenation to produce CO from CH_4_, the Ni–Mo*_x_* surface exhibits the lowest energy barrier for CH_2_ oxidation [22]. The uniform distribution of Mo doping, as observed in the formation of the NiMo alloy, improves the DRM reaction by providing more effective active sites. Furthermore, the adsorption energies of the intermediate species on the NiMo alloy are generally much higher than those on Ni(111) [20,22]. Similarly, Zhang et al. indicated that the adsorption energy of CH_4_ and CO_2_ on Ni(111) was approximately 0 eV, suggesting a weak interaction. However, the presence of Mo in the NiMo alloy facilitated stronger adsorption of both CH_4_ and CO_2_, which is favorable for the DRM reaction [19]. In PM-*x* catalysts, as shown in Figure 4c and Figure 4d, the highest conversion rates for CO_2_ and CH_4_ are 42.7%, which are generally lower than those observed for the OP-*x* series. The reduced performance is attributed to non-reduced Mo, which forms MoO_3_ crystals during the post-reduction process at 500 °C before the DRM reaction. In contrast, no MoO_3_ crystallites were observed in OP-1 and OP-2 samples, even after high-temperature DRM at 800 °C, consistent with their superior catalytic performance and supporting our interpretation. However, in OP-3 and higher Mo-loaded samples, rod-shaped MoO_3_ crystals begin to emerge. Elevated DRM temperatures likely accelerate the crystallization of initially amorphous MoO*_x_* species [29], leaving the excess Mo unintegrated into the NiMo alloy. This phenomenon is evident from the SEM images (Figure 5a,b), powder XRD patterns (Supporting Information File 1, Figure S7), and EDX mapping (Supporting Information File 1, Figure S8), which reveal the absence of NiMo alloy formation and the presence of chunky rod-shaped crystals.

To further support the hypothesis, we performed in situ diffuse reflectance infrared Fourier transform spectroscopy (DRIFTS) measurements on Ni/MgO, OP-1, OP-5, and PM-6 nanocatalysts (Figure 5c–f and Supporting Information File 1, Figure S9). Each sample was first exposed to pure CO_2_ (10 mL·min^−1^) at 750 °C for 30 min, followed by pure H_2_ (10 mL·min^−1^) to monitor the reduction of CO_2_-derived surface species and the formation of CO. Under H_2_ flow, the intensities of the adsorbed CO_2_-related bands gradually decreased. Meanwhile, characteristic bands associated with formate (≈1040 cm^−1^), bicarbonate (≈1090 cm^−1^), and formyl-type intermediates (≈1780 cm^−1^) increased transiently, reflecting their participation in the stepwise reduction process. In parallel, CO adsorption bands appeared, indicating the progressive conversion of CO_2_-derived surface intermediates into CO. Among all tested catalysts, OP-1 showed the fastest depletion of CO_2_-related bands and the clearest CO vibrational features (Figure 5d), indicating highly accessible and active sites for CO_2_ activation and reduction. In contrast, slower CO_2_ consumption in OP-5 (Figure 5e) indicates that excessive Mo leads to MoO_3_ crystallization, reducing CO_2_ activation and weakening synergistic effects between Ni and Mo. The PM-6 catalyst exhibited the poorest performance (Figure 5f) with persistent carbonate species and limited CO evolution, consistent with the inhibitory effect of surface MoO_3_ crystallites (Supporting Information File 1, Figure S6).

Additionally, OP-1 and OP-2 exhibited relatively higher H_2_/CO values while showing NOSCE behavior (Supporting Information File 1, Figure S10), whereas the H_2_/CO value decreased from that of OP-3 to OP-6. The relatively low H_2_/CO ratio (<1) can be attributed primarily to the fact that, under the high flow rate, the conversion did not reach completion. The residual CO_2_ likely reacted with H_2_ via the reverse water gas shift (RWGS) reaction on the Ni surfaces [30], thereby consuming H_2_ and lowering the H_2_/CO ratio. In addition, methane cracking (CH_4_ → C + 2H_2_) may also occur under DRM conditions, and the resulting carbon deposition can compete with the reforming reaction depending on the catalyst’s coke resistance [31]. However, given the incomplete CO_2_ conversion under these conditions, RWGS is expected to be the primary cause of the decreased H_2_/CO ratio. In contrast, all PM-*x* samples beyond PM-1 consistently maintained an H_2_/CO ratio below 0.5 throughout the reaction. These observations further confirm the cause of the catalytic performance degradation [22].

The powder XRD patterns of OP-*x* samples before the DRM reaction (Figure 2a) indicate the absence of MoO_3_ crystals. The observation is further corroborated by SEM images in Supporting Information File 1, Figure S11. The SEM images in Figure 5b show that the amount of crystalline MoO_3_ in OP-*x* increases with higher Mo loading at high temperatures, thereby covering the active sites of the NiMo catalyst and resulting in reduced catalytic activity. A theoretical study by Huang et al. suggested that the high coke resistance and conversion rate of the NiMo catalyst in DRM reactions are due to facile carbon elimination on the MoO*_x_*@Ni surface [22]. However, the experimental results indicate that MoO_3_ tends to form in the crystalline phase, especially at high temperatures, which does not contribute to the conversion efficiency. Instead, it decreases performance by covering the active sites of the catalyst. The theoretical study, we conclude, did not account for the crystallization possibility of MoO*_x_*.

**Figure 6:**
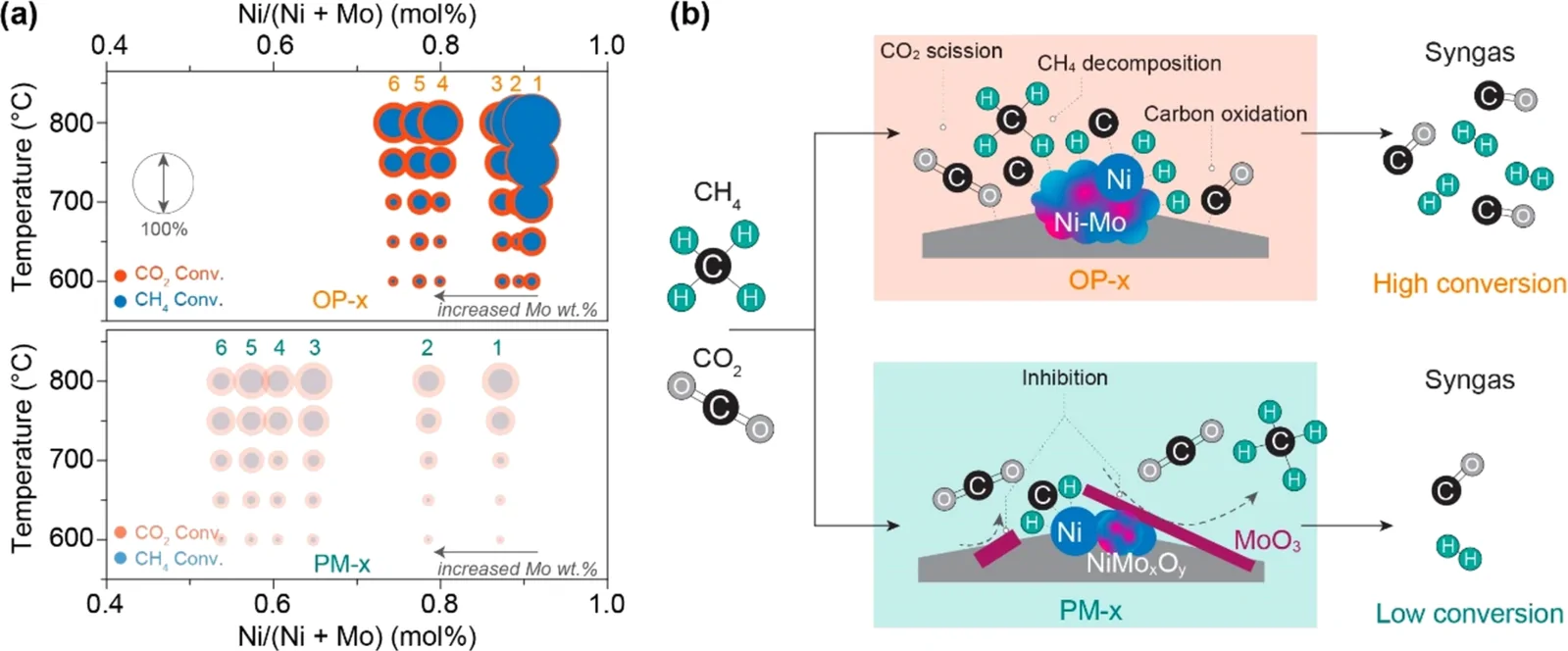
CO_2_ and CH_4_ conversion activities of (a) OP-*x* (top) and PM-*x* (bottom) samples with different Ni/(Ni+Mo) (mol %) ratios at various temperatures. (b) Schematic illustration of the fundamental CO_2_ and CH_4_ conversion mechanisms on NiMo alloy-rich OP-*x* and PM-*x* samples containing MoO_3_ crystals.

At the highest tested temperature, the samples with higher Ni content resulted in a higher conversion activity for both CO_2_ and CH_4_ (Figure 6). It is important to note that when preparing the OP-*x* and PM-*x* catalysts, the amounts of Ni and Mo added were intended to closely match the NiMo alloy formation ratio according to the Ni–Mo phase diagram (Supporting Information File 1, Figure S12). However, the actual ratios revealed by ICP-OES do not align well with the expected alloy formation ratios. In OP-6, the actual Ni/(Ni+Mo) ratio approached that of the Ni_4_Mo phase (Figure 6a); however, the sample exhibited the lowest catalytic activity due to the large amount of MoO_3_ crystals blocking the catalyst’s active sites. Interestingly, OP-1 and OP-2 showed the best catalytic performance, as they have more NiMo alloy formations and no observable chunky MoO_3_ crystals than others. These results highlight the complexity of the alloy formation process and suggest that factors beyond simple stoichiometric ratios play a critical role in determining catalytic activity.

In context, CH_4_ dissociation and CO_2_ activation are expected to proceed more efficiently in the OP-*x* samples (Figure 6). The high conversion rate arises from the abundance of active sites, such as NiMo alloy and Ni surfaces, which facilitate gas adsorption and desorption. In contrast, the PM-*x* sample contains MoO_3_ crystals that obstruct access to active sites, limiting gas adsorption and lacking the necessary active sites for efficient CO_2_ and CH_4_ activation [32].

Last, we investigated the coke resistance of Ni/MgO, Mo/MgO, SC MgO, and polycrystalline MgO to further support the NOSCE phenomenon and to clarify the role of Mo. After 48 h of DRM treatment (without inert gas dilution), thermogravimetric analysis (TGA) revealed that Mo/MgO (SC) exhibited the smallest weight loss (≈2.5 wt %), whereas Ni/MgO (SC), SC MgO, and polycrystalline MgO showed weight losses of 10, 8.5, and 14.5 wt %, respectively (Supporting Information File 1, Figure S13). These results indicate that Mo/MgO (SC) experienced the least carbon deposition among the investigated samples, supporting the notion that the NOSCE effect enables Mo species to occupy high-energy sites on SC MgO, which inherently possesses fewer defect sites, thereby enhancing coke resistance. As a result, carbon species are less likely to accumulate on Mo surfaces due to MoO*_x_* species having a unique MoO*_x_* ↔ MoC*_x_*O*_y_* redox cycle, which enhances carbon removal during DRM [19]. The results suggest that, although Ni–MoO*_x_* species do not exhibit high DRM activity, they play a critical stabilizing role by suppressing coke formation and preventing Ni oxidation, ultimately enhancing long-term catalyst durability.

## Conclusion

In summary, we synthesized a series of single-crystalline MgO-supported NiMo nanocatalysts with various Ni/Mo ratios using two different synthetic methods. The catalysts prepared through one-pot synthesis (OP) exhibited excellent catalytic performance, where the reduction of Mo to its metallic state facilitated NiMo alloy formation, achieving high DRM conversions. However, increasing the Mo content in the OP-*x* catalysts led to decreased catalytic activity due to the stabilization of Mo species as crystalline MoO_3_, which impedes the DRM reaction. In contrast, the post-modification (PM) samples exhibited a lower DRM activity, primarily because of the inefficient reduction of Mo. Because the PM-*x* samples have higher actual Mo loading than OP-*x*, MoO_3_ crystals formed during the post-reduction process, blocking the catalytic active sites and limiting the performance. These results challenge the theoretical assumption that MoO_3_ enhances DRM activity [22], demonstrating that excess Mo promotes an inactive phase that negatively affects catalytic conversions. Nevertheless, defective blockage could contribute to enhanced coke resistance. The NOSCE effect promotes the preferential coverage of high-surface-energy MgO sites (such as point defects and (111) or (110) planes) by Mo, highlighting its critical role in DRM.

The results provide valuable insights for the rational design of NiMo nanocatalysts for DRM applications. We conclude that it is essential to optimize the stoichiometric ratios and synthesis conditions when synthesizing bimetallic catalysts, mainly to prevent the formation of inactive phases such as MoO_3,_ while promoting NiMo alloy formation. Efficient reduction techniques and controlled precursor compositions are crucial for achieving active NiMo phases in catalysts. Although multiple species coexist in our catalyst system alongside the NiMo alloy, neither pure Ni nor Mo alone appears to have a detrimental effect on the reaction. Additionally, the synthesis method significantly influences the dispersion and interaction of Ni and Mo on the MgO support, ultimately affecting catalytic performance and stability. The study highlights the significance of optimizing synthesis strategies to achieve maximum DRM efficiency and to advance the understanding of bimetallic catalyst interactions.

## Experimental

### Materials

Nickel chloride hexahydrate (NiCl_2_∙6H_2_O, 97%) and ethanol (99%) were purchased from VWR. Ammonium molybdate tetrahydrate ((NH_4_)_6_Mo_7_O_24_∙4H_2_O, 99%) was obtained from Panreac Applichem. Sodium hydroxide (NaOH, 98%), ethylene glycol (99.5%), polyvinylpyrrolidone (PVP, *M*_w_ = 55,000), and hydrazine monohydrate (N_2_H_4_∙H_2_O, 98%) were purchased from Sigma-Aldrich. Single-crystal magnesium oxide (SC MgO) was provided by Mr. Wayne Dickinson and Mr. Matthew Bishop of the Dickinson Corporation (San Rafael, CA, USA). All chemicals were used as received without further purification.

### Nanocatalyst synthesis

One-pot (OP) synthesis of NiMo: Nickel chloride hexahydrate (1.7 mmol) and *x* mmol (*x* = 0.03, 0.06, 0.09, 0.12, 0.15, and 0.18 mmol, for OP-1 to OP-6) of ammonium molybdate tetrahydrate were dissolved in 24 mL of ethylene glycol. Polyvinylpyrrolidone (0.38 g) was added to the solution and stirred until complete dissolution was achieved. Subsequently, 0.9 g of SC MgO was added. The solution was then heated to 80 °C, and a hydrazine monohydrate/ethylene glycol solution (5 mL, 5.11 M) was introduced, causing the mixture to turn blue immediately. Sodium hydroxide/ethylene glycol solution (10 mL, 0.34 M) was added, and the mixture was maintained at 80 °C for 1 h. The solution was then washed with anhydrous ethanol and filtered using vacuum filtration. The filtered samples were dried overnight in a vacuum furnace at 80 °C. The collected fresh NiMo powder was then heat-treated at 500 °C under a 5% H_2_ flow for 3 h.

Post-modification (PM) of NiMo: Ni/MgO was initially prepared using above mentioned procedure, excluding the addition of Mo salt. Subsequently, a 250 mg·mL^−1^ solution of (NH_4_)_6_Mo_7_O_24_ in ethylene glycol was prepared. The desired volume of (NH_4_)_6_Mo_7_O_24_ solution (0.147–0.883 mL) was slowly added to 1 g of fresh Ni/MgO powder and thoroughly ground in a mortar with a pestle for approximately 15 min. The resulting mixture was dried overnight under a vacuum at 160 °C. The collected fresh NiMo powder was then heat-treated at 500 °C under a 5% H_2_ flow for 3 h.

### Nanocatalyst characterization

Powder X-ray diffraction (XRD) patterns were collected on a Bruker D8 ADVANCE diffractometer using Cu Kα radiation (λ = 1.5406 Å) over a 2θ range of 15°–80° at a scan rate of 5°·min^−1^. Scanning electron microscopy (SEM) and energy-dispersive X-ray spectroscopy (EDX) analyses were performed using a Carl Zeiss Merlin high-resolution scanning electron microscope equipped with an EDX detector. X-ray photoelectron spectroscopy (XPS) data were collected using a Thermo Scientific K-Alpha instrument with Al Kα radiation, operating at a scan rate of 1.0 eV per step and a spot size of 400 μm. Transmission electron microscopy (TEM) analysis was conducted using a Titan™ (FEI Company) operated at a beam energy of 300 keV and fitted with a Tridiem™ post-column energy filter (Gatan, Inc.). High-angle annular dark-field scanning transmission electron microscopy (HAADF-STEM) and elemental mapping were performed using a Talos f 200x (FEI Company). Inductively coupled plasma-optical emission spectrometry (ICP-OES) was performed on a Varian 720-ES ICP-optical emission spectrometer. In situ diffuse reflectance infrared Fourier transform spectroscopy (DRIFTS) measurements were carried out using a high-temperature DRIFTS cell equipped with a ZnSe window and connected to an FTIR spectrometer. Approximately 50 mg of catalyst was loaded into the sample holder, and the system was purged with high-purity helium (30 mL·min^−1^). The sample was first pretreated at 400 °C under flowing He for 1 h to remove any physisorbed moisture or residual volatile species. Subsequently, the temperature was gradually increased to 750 °C under He flow and stabilized. Once thermal equilibrium was reached, pure CO_2_ gas (10 mL·min^−1^) was purged and maintained for 30 min to allow for surface adsorption and reaction, while IR spectra were continuously recorded. Afterward, the gas feed was switched to pure H_2_ (10 mL·min^−1^) at the same temperature and held for another 30 min to observe the reduction of adsorbed CO_2_ species and formation of CO. Finally, the system was purged with He and allowed to cool to room temperature under inert conditions to complete the measurement cycle. Thermogravimetric analysis (TGA) was conducted at a ramping rate of 10 °C·min^−1^ using a TGA/DSC 3+ STAR^e^ System (METTLER TOLEDO Inc., Switzerland).

### Catalytic tests

Catalytic screening tests were conducted using a 16-channel Flowrence^®^ system (Avantium). 25 mg of OP-*x* (or PM-*x*) (*x* = 1–6) with SiC frits (400 μL) were loaded into the reactors, the 300 mm long quartz tubes (I.D. 2 mm, O.D. 3 mm), before being inserted into a furnace. One reactor, filled with SiC frits (400 μL), was used as a blank. A controlled 10 mL·min^−1^ of mixed feed (CH_4_/CO_2_/N_2_ = 2:2:6) was introduced in each reactor with 0.7 mL·min^−1^ of He as an internal standard. The weight hourly space velocity (WHSV) was 25,680 mL·g^−1^·h^−1^ for each reactor. Before feeding the reaction mixture, all samples were pretreated in a pure H_2_ atmosphere for 3 h at 500 °C. Then, the activity was screened from 600 to 800 °C, and all products were carefully analyzed by gas chromatography (GC, Agilent 7890 B), ensuring the highest level of accuracy. The conversions of CO_2_ and CH_4_ were calculated using Equation 2 and Equation 3.


[2] $$X_{{\text{CO}}_{2}}=\frac{{\text{CO}}_{2\text{ in}}/{\text{He}}_{\text{in}}-{\text{CO}}_{2\text{ out}}/{\text{He}}_{\text{out}}}{{\text{CO}}_{2\text{ in}}/{\text{He}}_{\text{in}}}\times 100\left(\%\right)$$

[3] $$X_{{\text{CH}}_{4}}=\frac{{\text{CH}}_{4\text{ in}}/{\text{He}}_{\text{in}}-{\text{CH}}_{4\text{ out}}/{\text{He}}_{\text{out}}}{{\text{CH}}_{4\text{ in}}/{\text{He}}_{\text{in}}}\times 100\left(\%\right)$$

## Supporting Information

File 1 Additional figures and table.

## Data Availability

Data generated and analyzed during this study is available from the corresponding author upon reasonable request.
