# Supplementary material for: Optimization of NiMo alloy nanoparticles for catalytic dry reforming of methane
Source: Beilstein J Nanotechnol. 2026 Aug 31;17:1212–22. doi: 10.3762/bjnano.17.84 (PMC13575340; doi:10.3762/bjnano.17.84)
Supplement: Supplementary file 1 [file Beilstein_J_Nanotechnol-17-1212-s001.pdf]

BEILSTEIN JOURNAL OF NANOTECHNOLOGY

Supporting Information

for

Optimization of NiMo alloy nanoparticles for catalytic dry reforming of methane

Azimet A. Karluk, Javeed Mahmood, Seok-Jin Kim and Cafer T. Yavuz

Beilstein J. Nanotechnol. **2026,** *17,* 1212–1222. doi:10.3762/bjnano.17.84

Additional figures and table

License and Terms: This is a supporting information file under the terms of the Creative Commons Attribution License (https://creativecommons.org/licenses/by/4.0). Please note that the reuse, redistribution and reproduction in particular requires that the author(s) and source are credited and that individual graphics may be subject to special legal provisions.
The license is subject to the *Beilstein Journal of Nanotechnology* terms and conditions: (https://www.beilstein-journals.org/bjnano/terms)

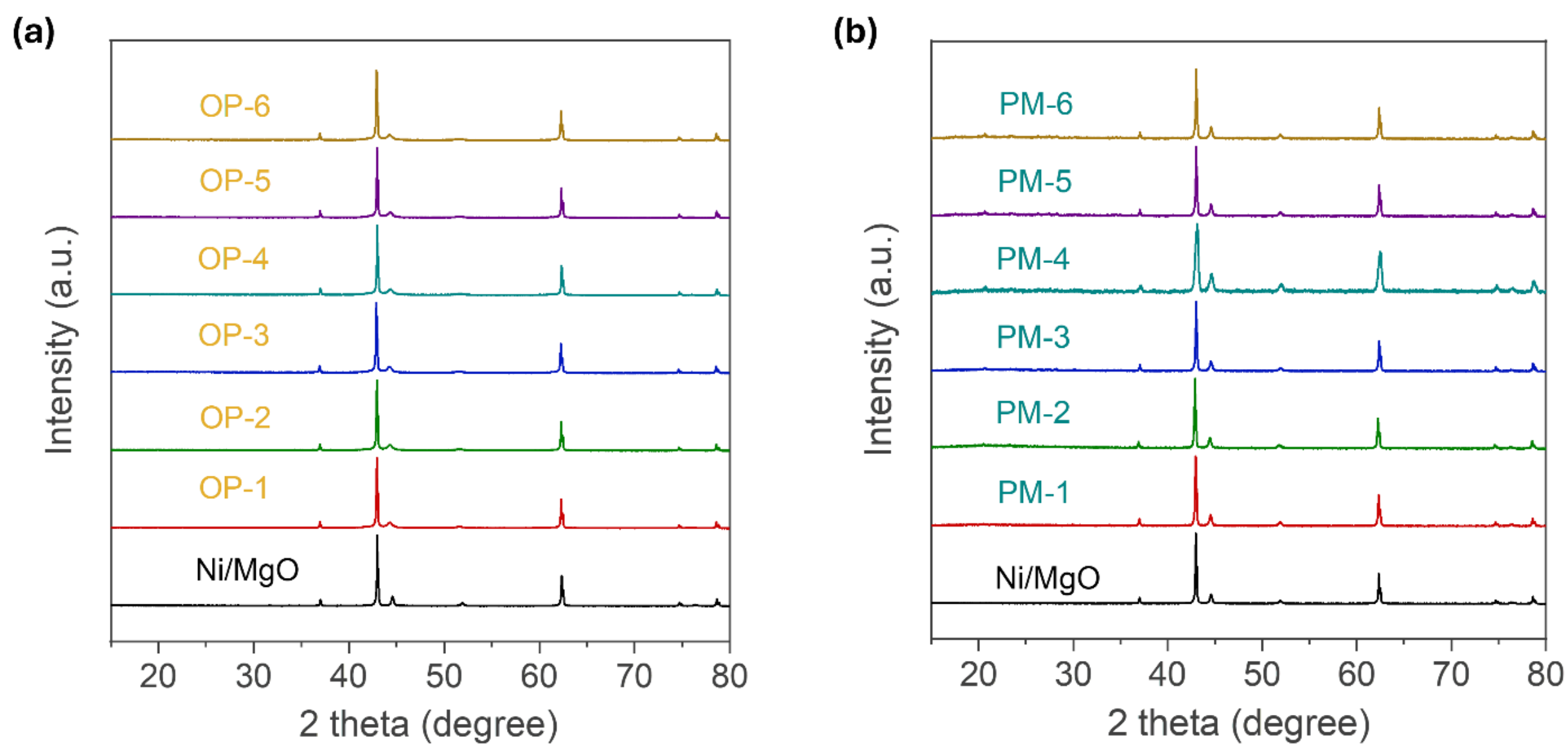

Figure S1: Powder XRD patterns of (a) OP-*x* and (b) PM-*x*, before DRM reaction.

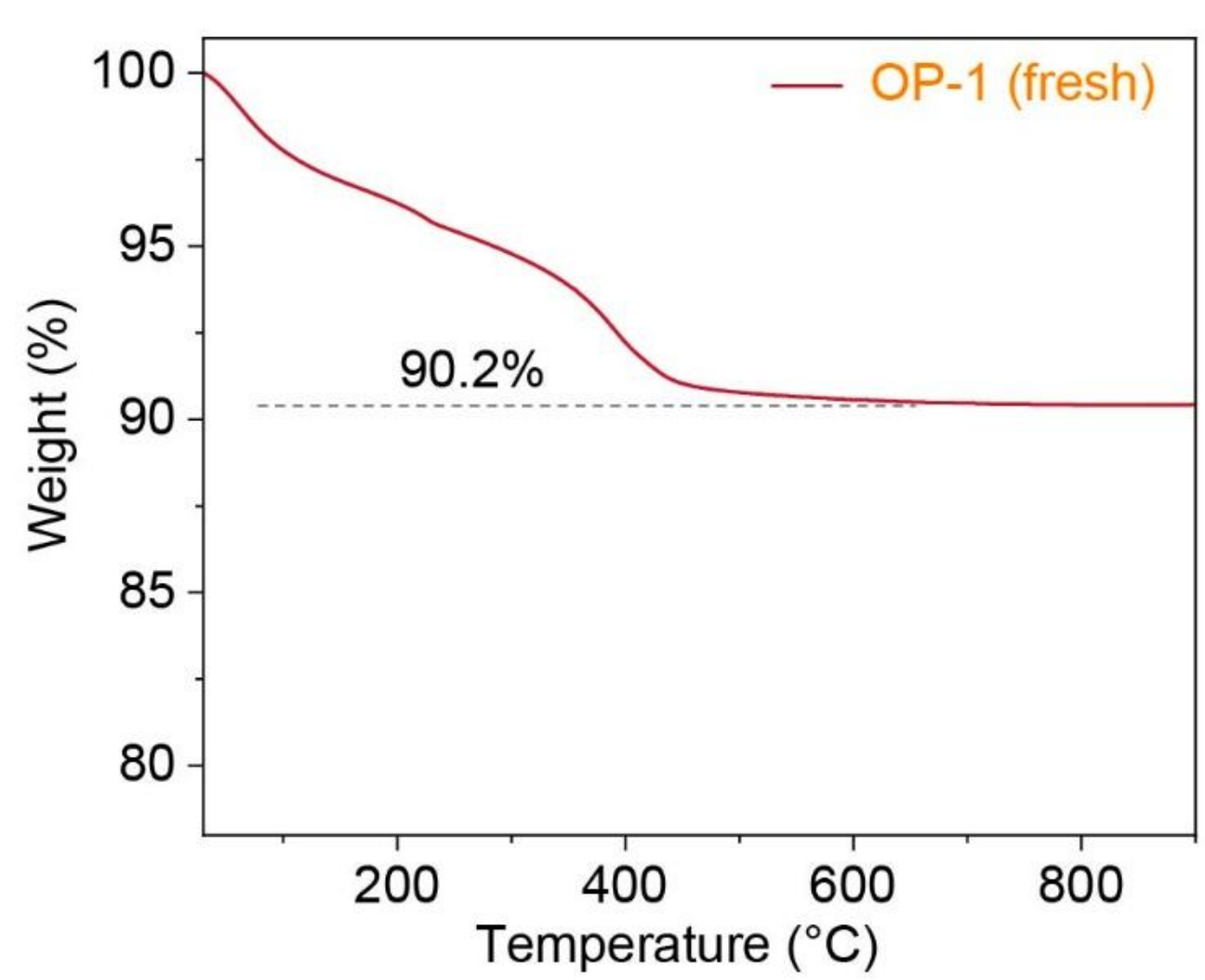

Figure S2: Thermogravimetric analysis (TGA) profile of fresh OP-1 recorded under N_2 flow (20 mL·min^{-1}), with a heating rate of 10 °C·min^{-1}. The total weight loss of ≈9.8 wt % is attributed to the removal of adsorbed species and decomposition of residual surface functionalities.

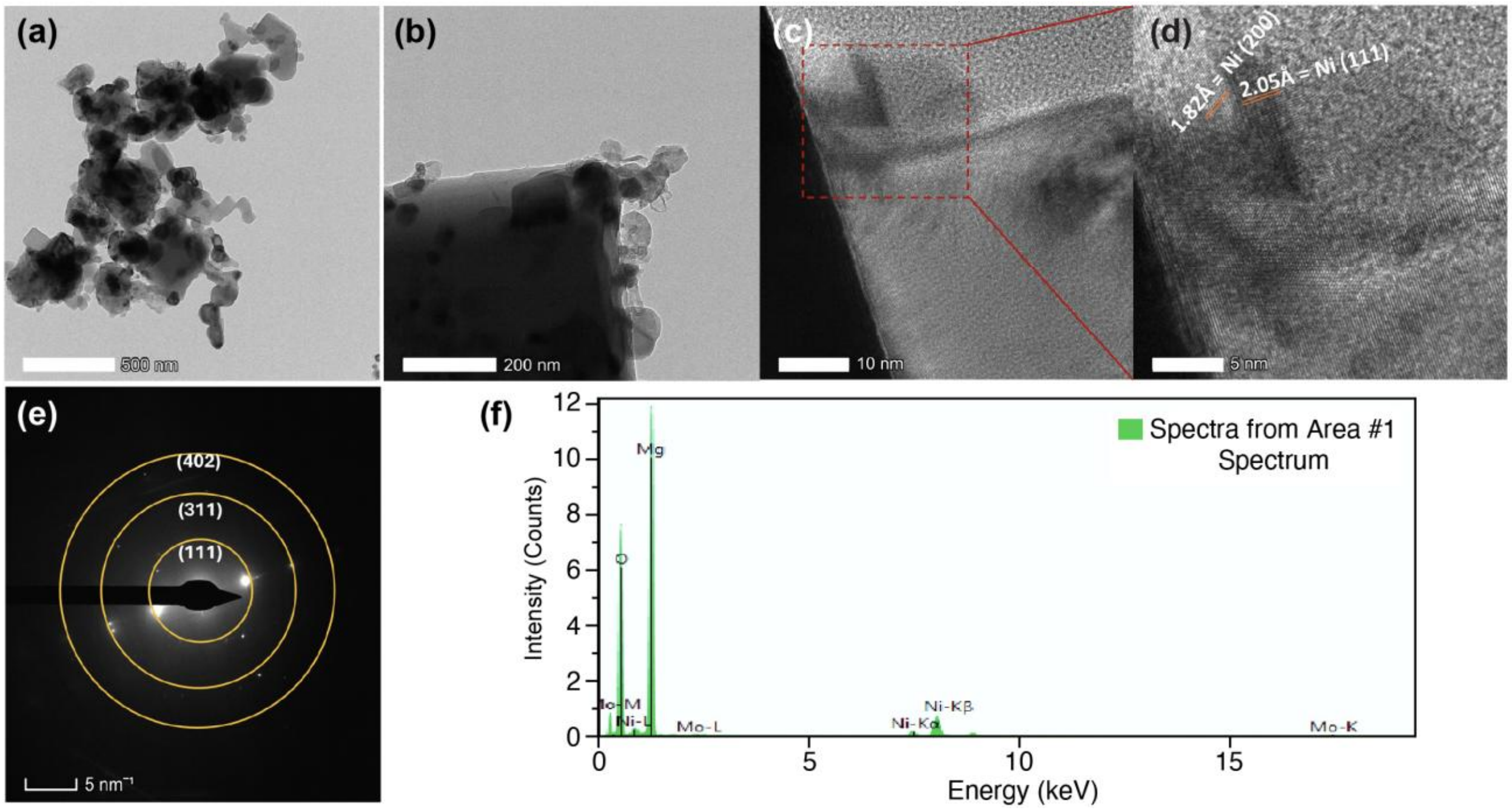

Figure S3: (a-b) TEM images, (c-d) High resolution TEM (HR-TEM) images, (e) Selected area electron diffraction (SAED) pattern, and (f) energy-dispersive X-ray (EDX) spectra of OP-2. The HR-TEM image reveals clear lattice fringes consistent with the SAED patterns. EDS confirms the elemental composition of the sample.

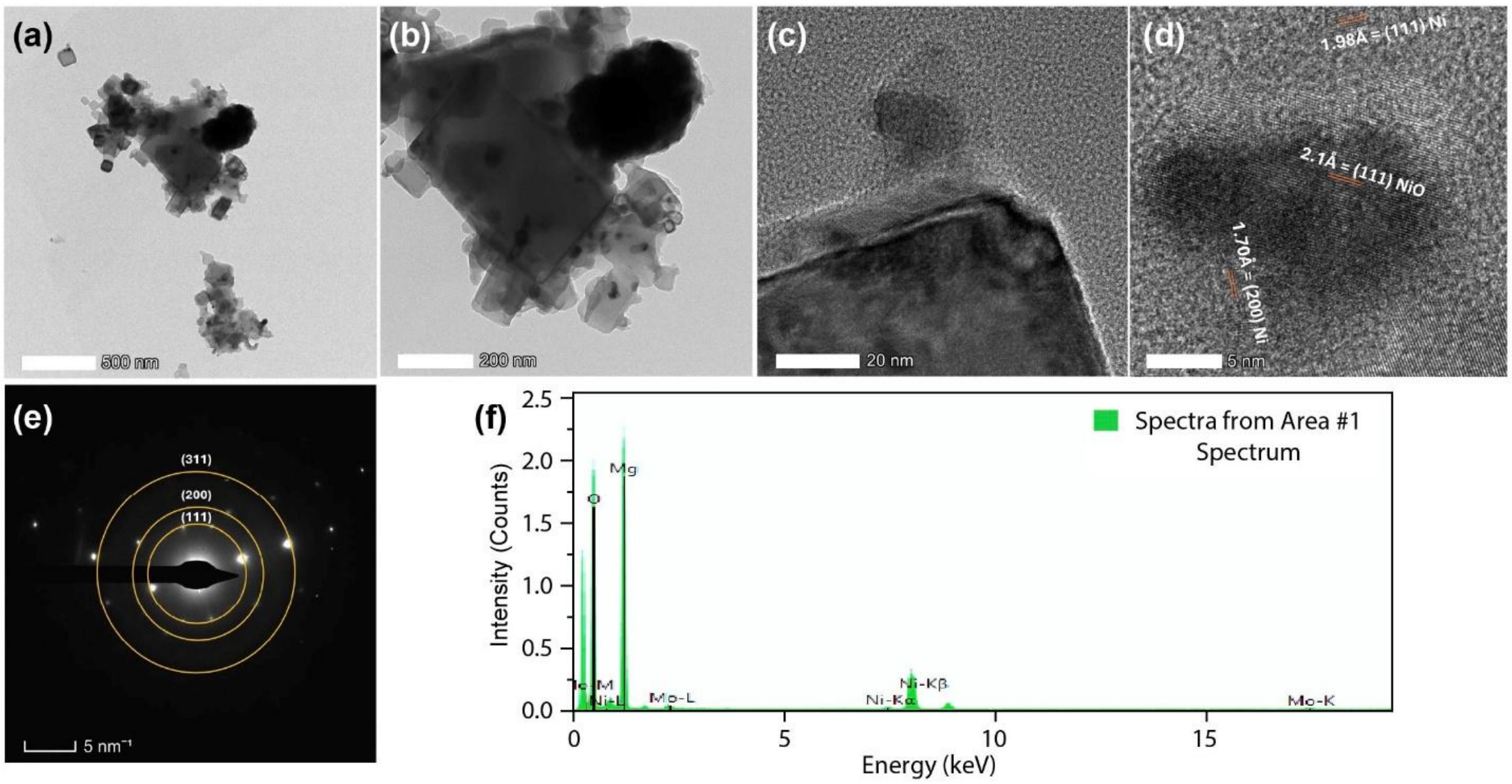

Figure S4: (a-b) TEM images, (c-d) High resolution TEM (HR-TEM) images, (e) Selected area electron diffraction (SAED) pattern, and (f) Energy-dispersive X-ray (EDX) spectra of PM-2. The HR-TEM image reveals clear lattice fringes consistent with the SAED patterns. EDS confirms the elemental composition of the sample.

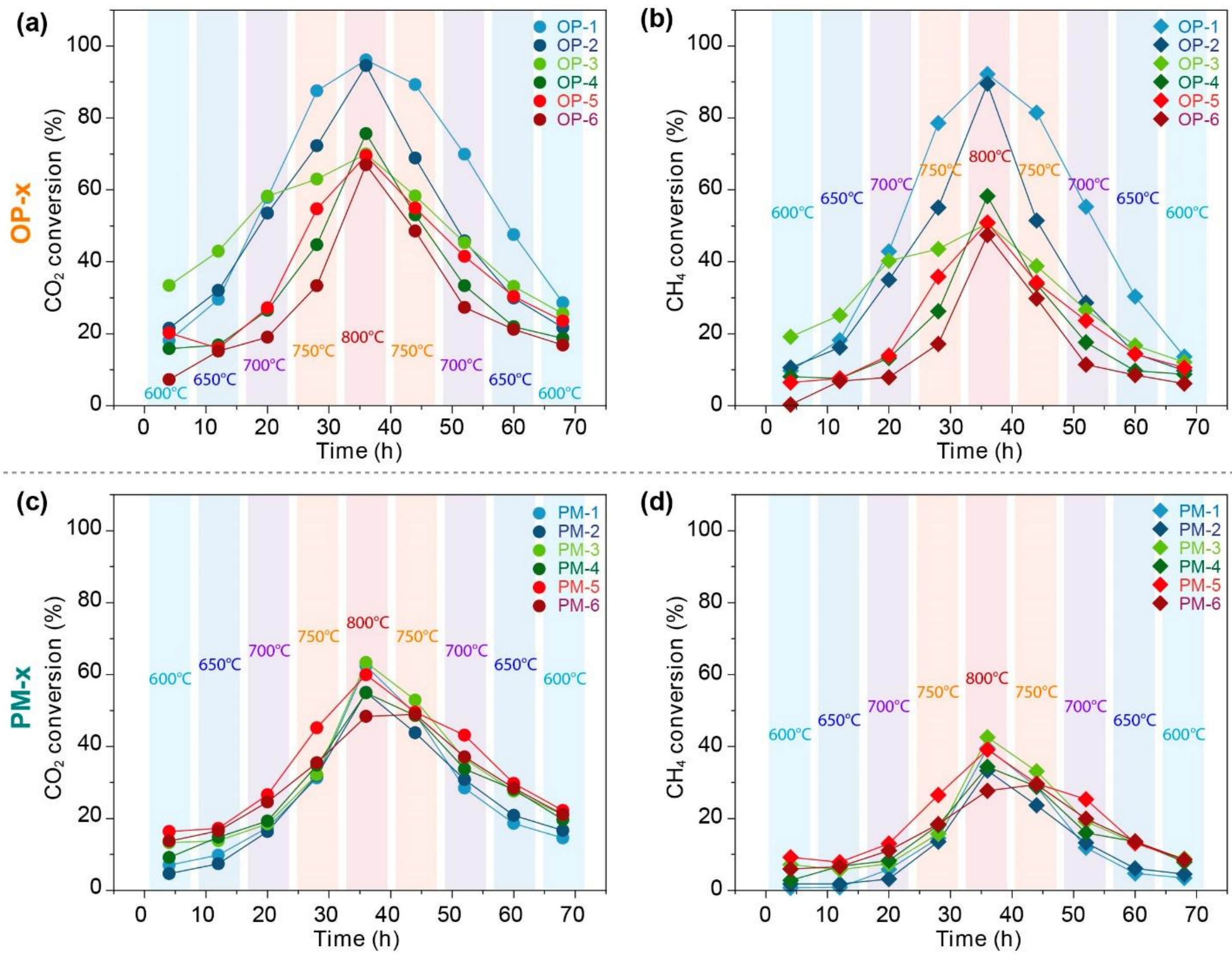

Figure S5: CO_2 and CH_4 conversions of (a-b) OP-*x* and (c-d) PM-*x* at different temperatures (Reaction conditions: 1 bar, 25 mg OP-*x* or PM-*x*, $CH_4/CO_2/N_2$ = 2:2:6, weight hourly space velocity (WHSV) = 25,680 mL·g^{-1}·h^{-1}).

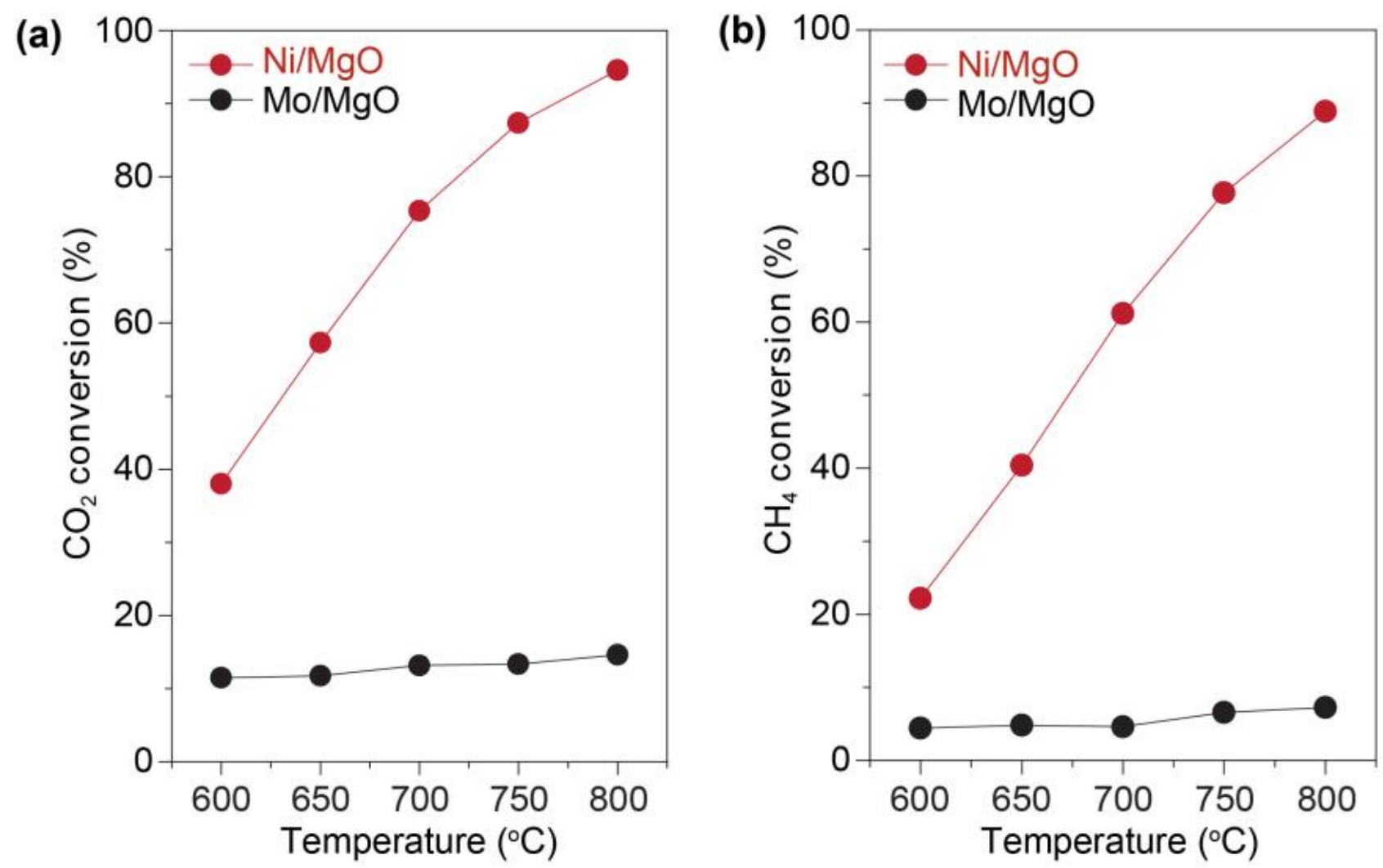

Figure S6: Comparison of CO_2 (a) and CH_4 conversions (b) of only Ni and only Mo on MgO at different temperatures (Reaction conditions: 1 bar, 25 mg, $CH_4/CO_2/N_2$ = 2:2:6, weight hourly space velocity (WHSV) = 25,680 mL·g^{-1}·h^{-1}).

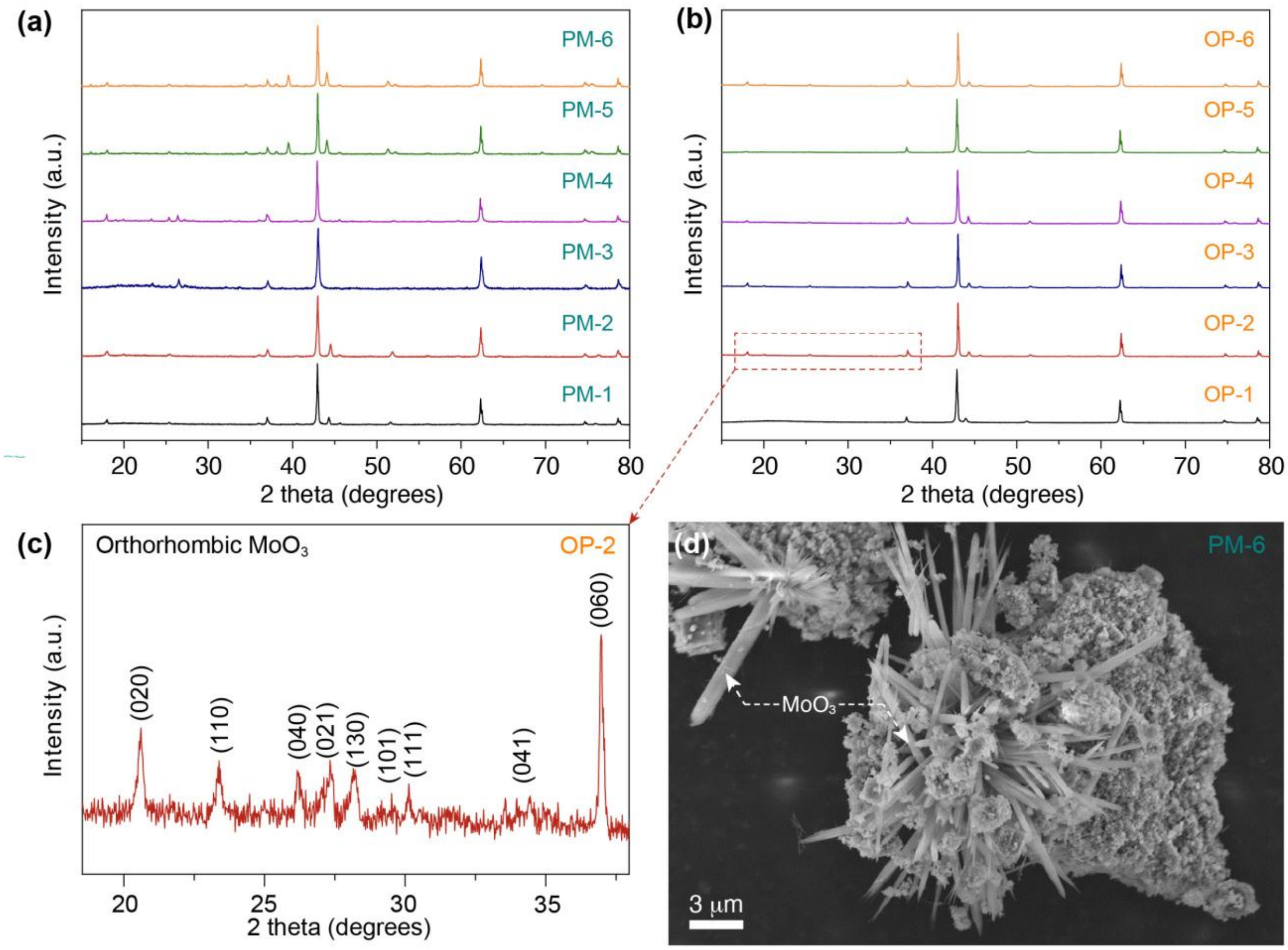

Figure S7: PXRD patterns of (a) PM-*x* before DRM and (b) OP-*x* samples after DRM reaction; (c) Enlarged XRD patterns which indicate the orthorhombic phase of MoO_3 (JCPDS-05-0508) crystal from OP-2; (d) SEM images of PM-6 after DRM reaction.

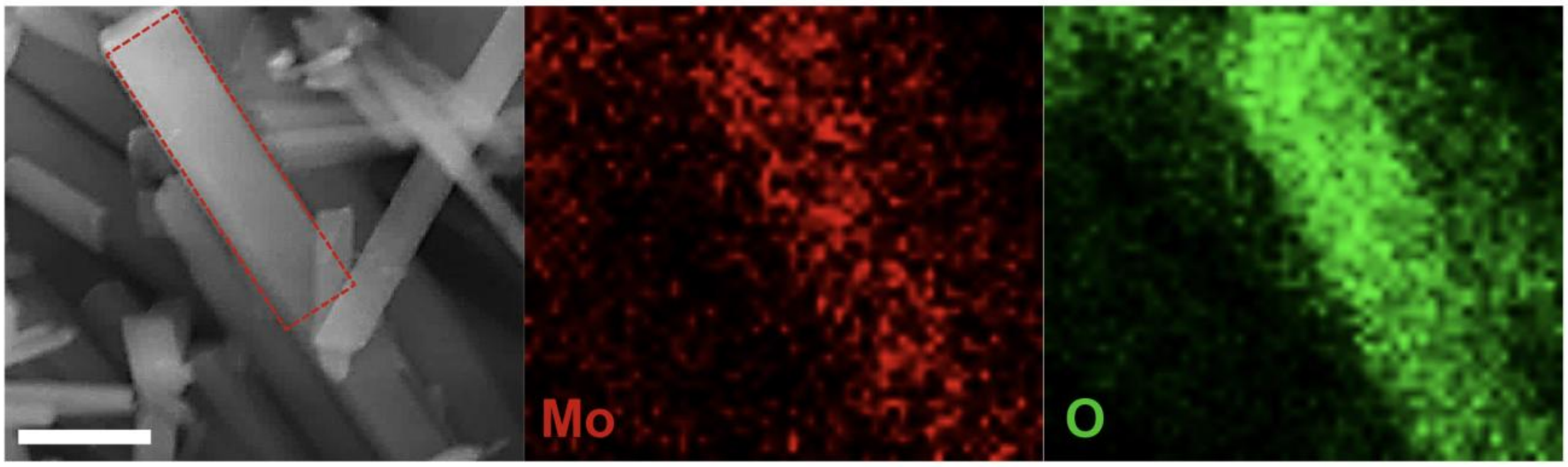

Figure S8: SEM image of rod-shaped MoO_3 crystal and EDS map for PM-6 (Scale bar: 1 μm, Red: Mo, Green: O).

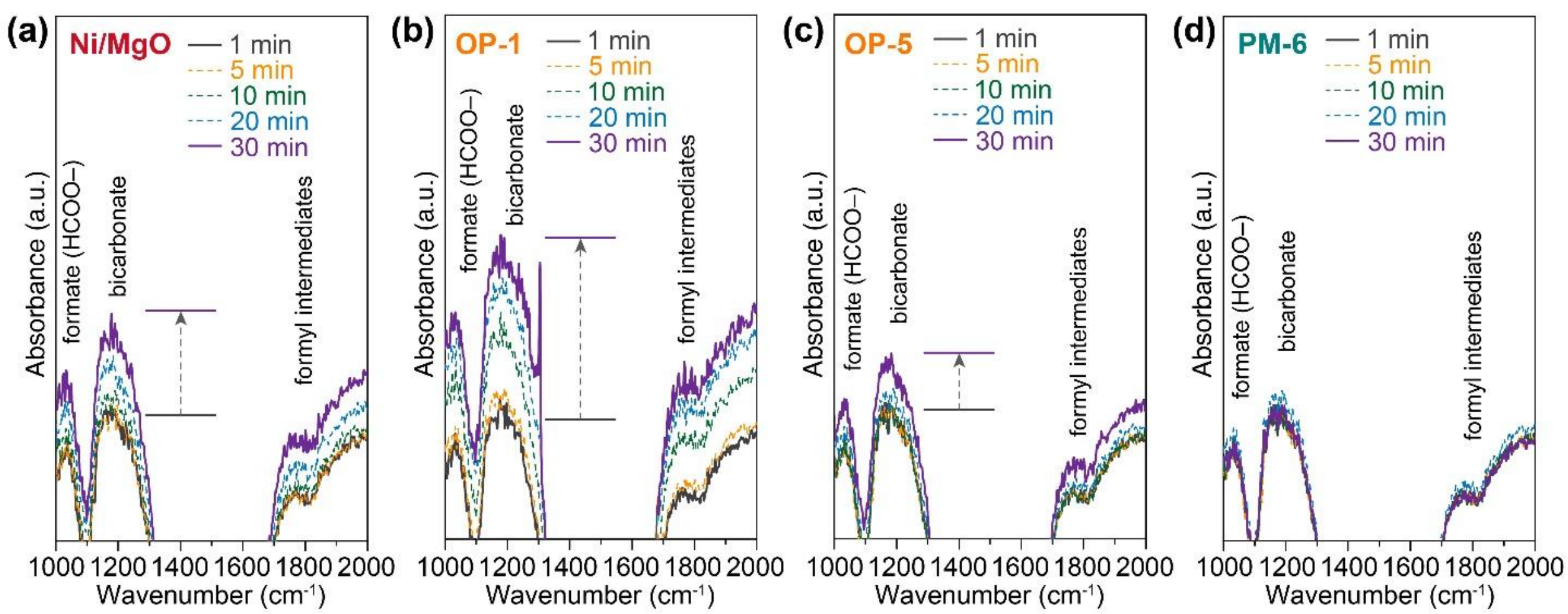

Figure S9: In situ DRIFT spectra collected during CO_2 reduction at 750 °C on Ni/MgO, OP-1, OP-5, and PM-6, respectively. The spectra were acquired sequentially under pure CO_2 flow (99.999%, 10 mL·min^{-1}, 30 min) followed by pure H_2 flow (99.999%, 10 mL·min^{-1}, 30 min).

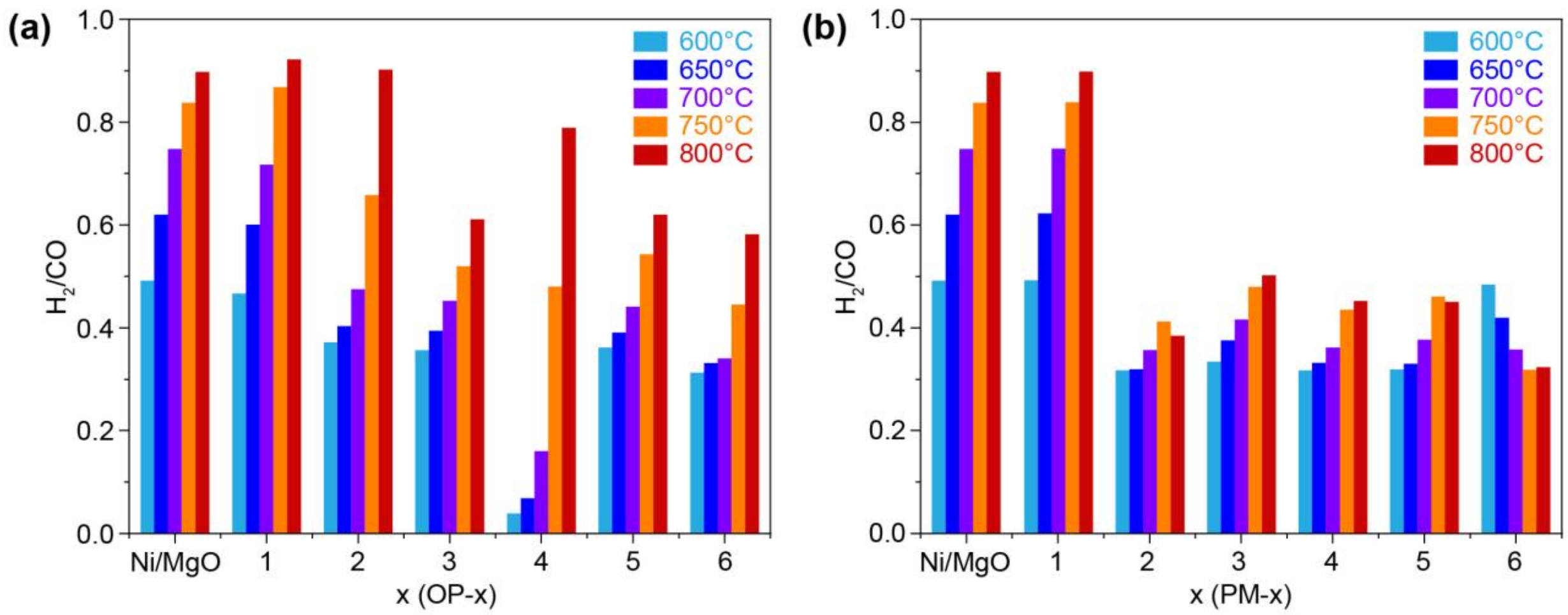

Figure S10: H_2 to CO ratios of OP-*x* samples (a) and PM-*x* samples (b) at different temperatures from 600 to 800 °C (Reaction conditions: 1 bar, 25 mg OP-*x* or (PM-*x*), $CH_4/CO_2/N_2$ = 2:2:6, Weight hourly space velocity (WHSV): 25,680 mL·g^{-1}·h^{-1}).

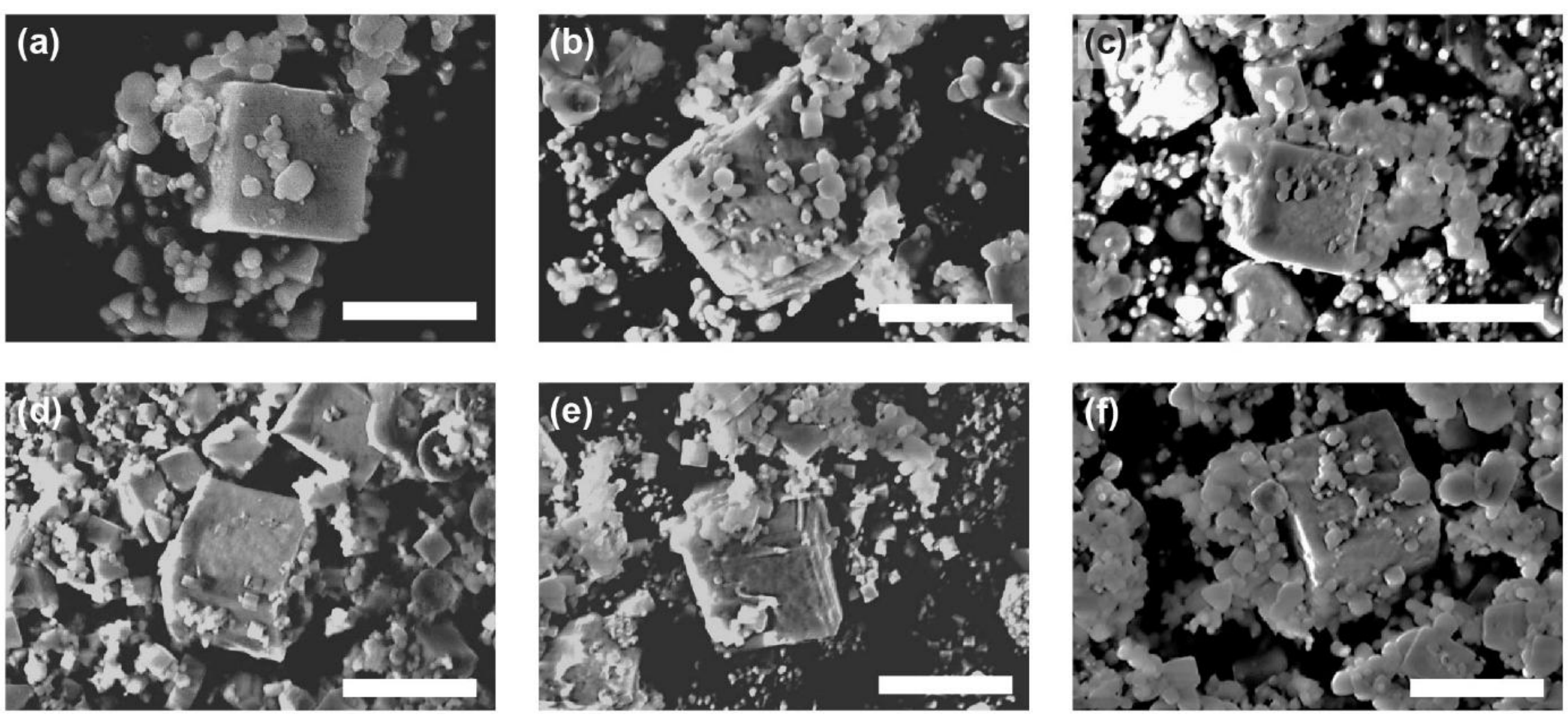

Figure S11: SEM images of OP-*x* (*x* = 1–6) before DRM (scale bar 1 μm). OP-1 to 6 all represent (a), (b), (c), (d), (e), and (f) in that order.

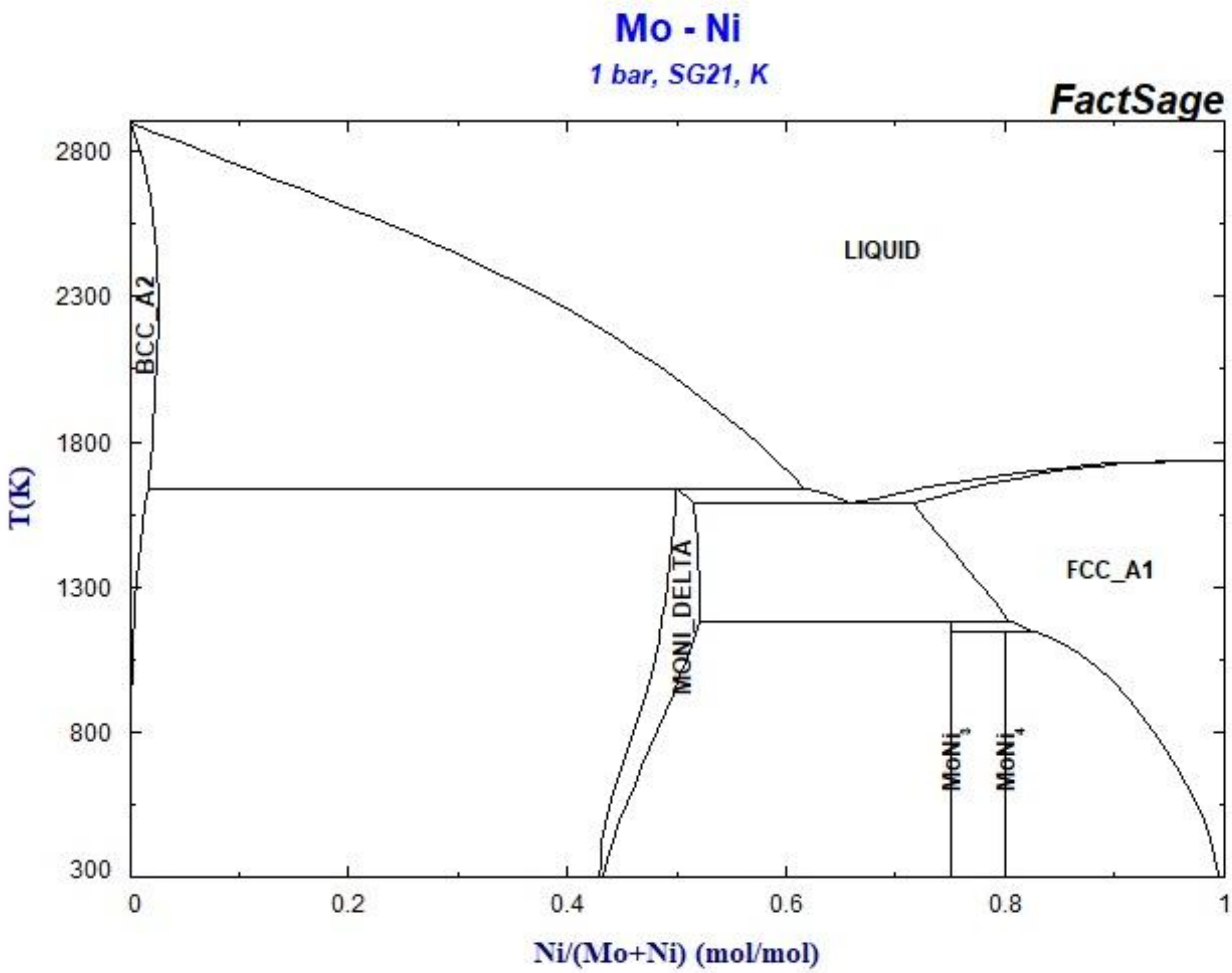

Figure S12: Alloy phase diagrams of Ni-Mo, from FactSage® [1] thermochemical values collection on SGTE 2022 alloy database, https://www.crct.polymtl.ca/fact/phase_diagram.php?file=Mo-Ni.jpg&dir=SGTE2022.

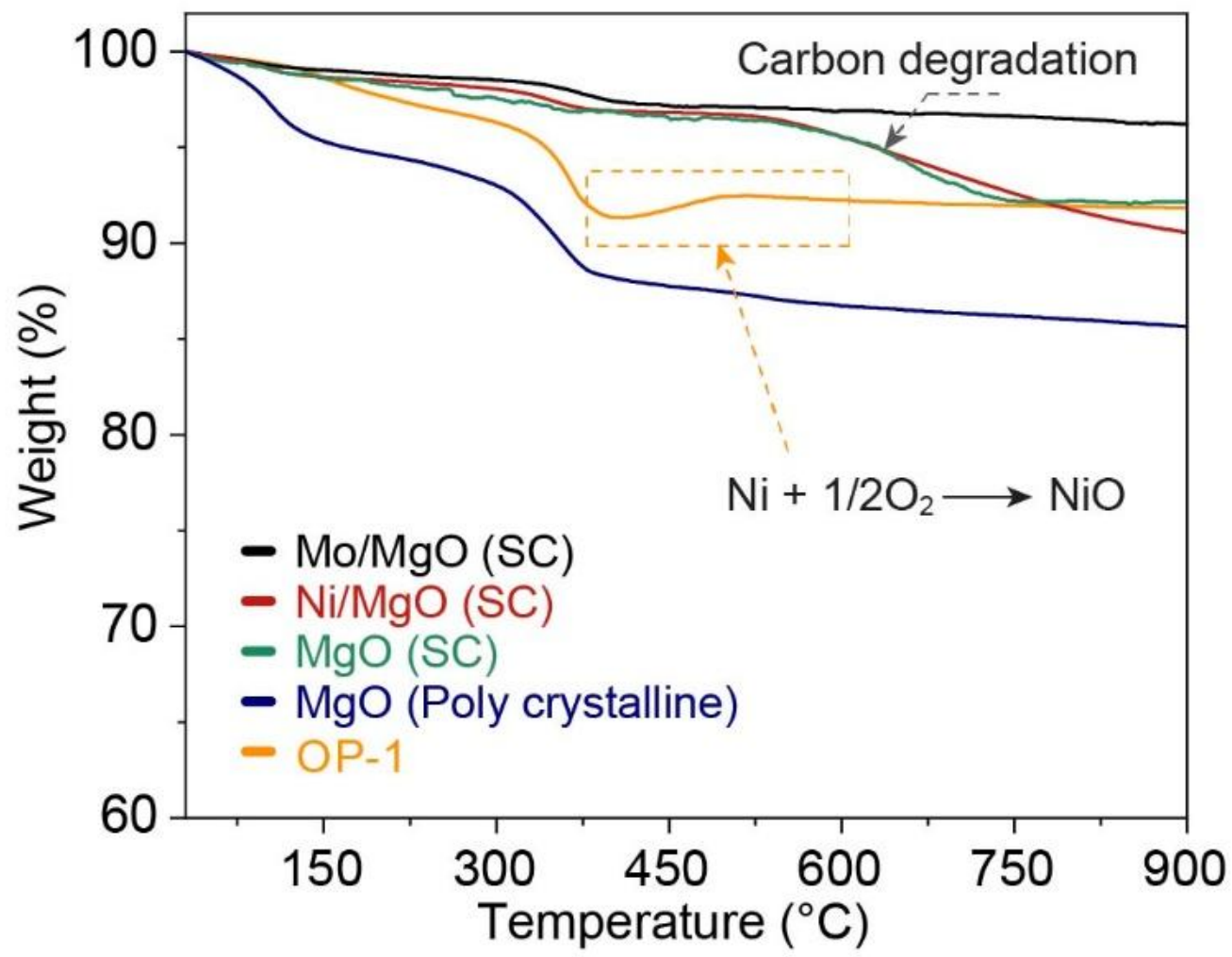

Figure S13: TGA analysis of Ni/MgO (single crystalline, SC), Mo/MgO (SC), MgO (SC), and polycrystalline MgO was conducted under air flow with a temperature ramp of 10 °C·min^{-1}. Samples were analyzed after exposure to harsh DRM conditions (10 mL·min^{-1} of CH_4 and CO_2 flow, no inert gas, for 48 hours at 800 °C).

Table S1: ICP-OES analysis results of OP-*x* and PM-*x* samples. The table lists the expected Ni and Mo loadings (wt %), together with the actual metal contents measured for catalysts synthesized *via* the one-pot (OP) method and after post-modification (PM).

**Catalysts**	**Expected metal loading (wt %)**		**One-pot synthesis (OP) ICP (wt %)**		**Post-modification (PM) ICP (wt %)**	
	**Ni**	**Mo**	**Ni**	**Mo**	**Ni**	**Mo**
**NiMo-1**	8.80	1.75	7.01	1.14	6.79	1.61
**NiMo-2**	8.63	3.45	6.70	1.30	6.67	2.98
**NiMo-3**	8.47	5.08	6.69	1.60	6.48	5.01
**NiMo-4**	8.31	6.66	5.80	2.40	6.32	6.51
**NiMo-5**	8.16	8.24	5.70	2.68	6.30	7.62
**NiMo-6**	8.02	9.63	5.04	2.83	6.27	8.80

Reference

1. Bale, C. W.; Chartrand, P.; Degterov, S.; Eriksson, G.; Hack, K.; Mahfoud, R. B.; Melançon, J.; Pelton, A.; Petersen, S. *Calphad* **2002**, *26* (2), 189-228.
